# Digital Mobility Assessment for Regulatory and Clinical Endorsement in Hip Fracture Patients

**DOI:** 10.1093/geroni/igab046.3179

**Published:** 2021-12-17

**Authors:** Carl-Philipp Jansen, Kristin Taraldsen, Hubert Blain, Clemens Becker

**Affiliations:** 1 Robert-Bosch-Hospital Stuttgart, Karlsruhe, Baden-Wurttemberg, Germany; 2 Norwegian University of Science and Technology, Trondheim, Sor-Trondelag, Norway; 3 Centre Hospitalier Universitaire de Montpellier, Montpellier, Aquitaine, France; 4 Robert-Bosch-Hospital, Stuttgart, Baden-Wurttemberg, Germany

## Abstract

Hip fracture is the most frequent non-intentional injury of older persons leading to hospital admission in Europe and North America. Until recently, in regulatory submissions no attention was given to patients’ mobility after sustaining/recovering from a hip fracture. To better evaluate efficacy and effectiveness of new drugs and treatments, it is necessary to develop mobility biomarkers since failure to recover and regain pre-fracture mobility is considered the single most important disability symptom experienced by hip fracture patients, often leading to care home admission. However, regularly used measures of mobility capacity are not representative of individuals’ performance in real life, intermittent in nature, and require visiting study centers. Digital technology has the potential to revolutionize mobility assessment in a real-life setting. With this presentation we build a case for a valid solution for real-world digital mobility assessment in hip fracture patients as carried out in the “Mobilise-D” clinical validation study.

